# Efficacy of a Low-Cost Bubble CPAP System in Treatment of Respiratory Distress in a Neonatal Ward in Malawi

**DOI:** 10.1371/journal.pone.0086327

**Published:** 2014-01-29

**Authors:** Kondwani Kawaza, Heather E. Machen, Jocelyn Brown, Zondiwe Mwanza, Suzanne Iniguez, Al Gest, E. O'Brian Smith, Maria Oden, Rebecca R. Richards-Kortum, Elizabeth Molyneux

**Affiliations:** 1 Department of Pediatrics, College of Medicine, Queen Elizabeth Central Hospital, Blantyre, Malawi; 2 Department of Pediatrics, Baylor College of Medicine, Houston, Texas, United States of America; 3 Department of Bioengineering, Rice University, Houston, Texas, United States of America; 4 Department of Respiratory Care, Texas Children's Hospital, Houston, Texas, United States of America; Hôpital Robert Debré, France

## Abstract

**Background:**

Respiratory failure is a leading cause of neonatal mortality in the developing world. Bubble continuous positive airway pressure (bCPAP) is a safe, effective intervention for infants with respiratory distress and is widely used in developed countries. Because of its high cost, bCPAP is not widely utilized in low-resource settings. We evaluated the performance of a new bCPAP system to treat severe respiratory distress in a low resource setting, comparing it to nasal oxygen therapy, the current standard of care.

**Methods:**

We conducted a non-randomized convenience sample study to test the efficacy of a low-cost bCPAP system treating newborns with severe respiratory distress in the neonatal ward of Queen Elizabeth Central Hospital, in Blantyre, Malawi. Neonates weighing >1,000 g and presenting with severe respiratory distress who fulfilled inclusion criteria received nasal bCPAP if a device was available; if not, they received standard care. Clinical assessments were made during treatment and outcomes compared for the two groups.

**Findings:**

87 neonates (62 bCPAP, 25 controls) were recruited. Survival rate for neonates receiving bCPAP was 71.0% (44/62) compared with 44.0% (11/25) for controls. 65.5% (19/29) of very low birth weight neonates receiving bCPAP survived to discharge compared to 15.4% (1/13) of controls. 64.6% (31/48) of neonates with respiratory distress syndrome (RDS) receiving bCPAP survived to discharge, compared to 23.5% (4/17) of controls. 61.5% (16/26) of neonates with sepsis receiving bCPAP survived to discharge, while none of the seven neonates with sepsis in the control group survived.

**Interpretation:**

Use of a low-cost bCPAP system to treat neonatal respiratory distress resulted in 27% absolute improvement in survival. The beneficial effect was greater for neonates with very low birth weight, RDS, or sepsis. Implementing appropriate bCPAP devices could reduce neonatal mortality in developing countries.

## Introduction

Severe respiratory distress is a common and serious complication of premature birth, neonatal pneumonia, and neonatal sepsis, which together account for over one-half of all neonatal deaths globally [1]. More than 50% of babies born at ≤31 weeks of gestation will develop respiratory distress syndrome (RDS) [2]. Respiratory distress is associated with over 80% of cases of neonatal pneumonia [3] and most cases of neonatal sepsis [4].

In the developed world, respiratory support is provided to neonates using either mechanical ventilation or Continuous Positive Airway Pressure (CPAP). Unfortunately, ventilators and CPAP machines are too expensive and technically complex for many resource-limited settings [5]. As a result, respiratory illness remains one of the most common causes of neonatal death in the developing world.

CPAP is a gentle and effective tool to treat even preterm and low birth weight infants in respiratory distress [6]–[11]. Well-resourced hospitals use ventilators, stand-alone CPAP devices, or tubing, wall air and oxygen to set up CPAP at the bedside [8]. In bubble CPAP (bCPAP), pressure is safely regulated by submerging the end of the tubing in a bottle of water. The depth of water determines the pressure in the system. This pressure helps recruit alveoli and increase functional residual lung capacity [12], thus lowering the baby's work of breathing. The result is better compliance, reduced airway resistance, conservation of surfactant, and stabilized chest and diaphragm [13]. Bubble CPAP has been used in developed countries for decades [8]. It reduces morbidity [6] and mortality [5], [8], as well as the need for mechanical ventilation [6], [7], [9]–[11], [14], [15]. It can be administered by trained nurses [14], [16], and is safer than mechanical ventilation [6], [14]. It also reduces hospital stay [6] and up-referrals [17]. As a result CPAP is increasingly used as a first choice for ventilatory support in tertiary centres [9], [14], [18].

A number of observational studies have shown that CPAP can be safely implemented in low-resource settings using commercially available devices designed for high-resource settings [14], [19]–[22]. Unfortunately, the cost and complexity of currently available CPAP devices is prohibitive for many low-resource settings.

We recently developed a novel, low-cost bCPAP system for low-resource settings that can be assembled for approximately $350, a fifteen-fold cost reduction compared to the average stand-alone CPAP. The device delivers the same therapeutic flow and pressure as bCPAP systems used in high-resource settings [23]. Here, we report a study to evaluate the efficacy of the new low-cost bCPAP system in improving survival in newborns with severe respiratory distress at Queen Elizabeth Central Hospital in Malawi. Malawi has a population of 15.4 million [24], with a preterm birth rate of 18% [25], neonatal mortality rate of 27 and infant mortality rate of 53 per 1000 live births [26]. The GDP is $805 per capita and 73.9% of the population live on less than $1.25 per day [27]. Queen Elizabeth Central Hospital is the main referral hospital in the southern region of the country; each year, about 10,000 babies are delivered at the hospital and over 3,000 are admitted to the neonatal ward [28].

## Methods

### Ethics Statement

The study protocol was approved by the University of Malawi College of Medicine Research and Ethics Committee (P.05/11/1079) and the Institutional Review Boards at Baylor College of Medicine (H-29059) and Rice University (11-198F) prior to study initiation. Written informed consent was obtained from parents or legal guardians before enrolling patients in the study.

### Participants

This prospective, non-randomized controlled study, conducted at Queen Elizabeth Central Hospital, evaluated the efficacy and safety of a novel, low-cost bCPAP device to treat neonatal respiratory illness in a low-resource setting. In Malawi, nasal oxygen from an oxygen concentrator is the standard of care to treat respiratory insufficiency in neonates. The study evaluated whether bCPAP treatment improves survival for neonates with respiratory illness compared to standard nasal oxygen.

Infants admitted to the neonatal ward with severe respiratory distress as defined by the presence of severe chest in-drawing, central cyanosis, wheezing, grunting, or nasal flaring were eligible to participate. In addition, patients had to weigh 1,000 grams or more at enrollment, be breathing spontaneously, and be neurologically viable. The treating clinician also had to deem bCPAP appropriate treatment. Patients who presented with cleft palate, trachea-oesophageal fistula, diaphragmatic hernia, severe cardiac instability, and severe birth asphyxia were not eligible. Subjects were identified by clinicians working in the neonatal ward.

### Procedures

**Figure 1:**
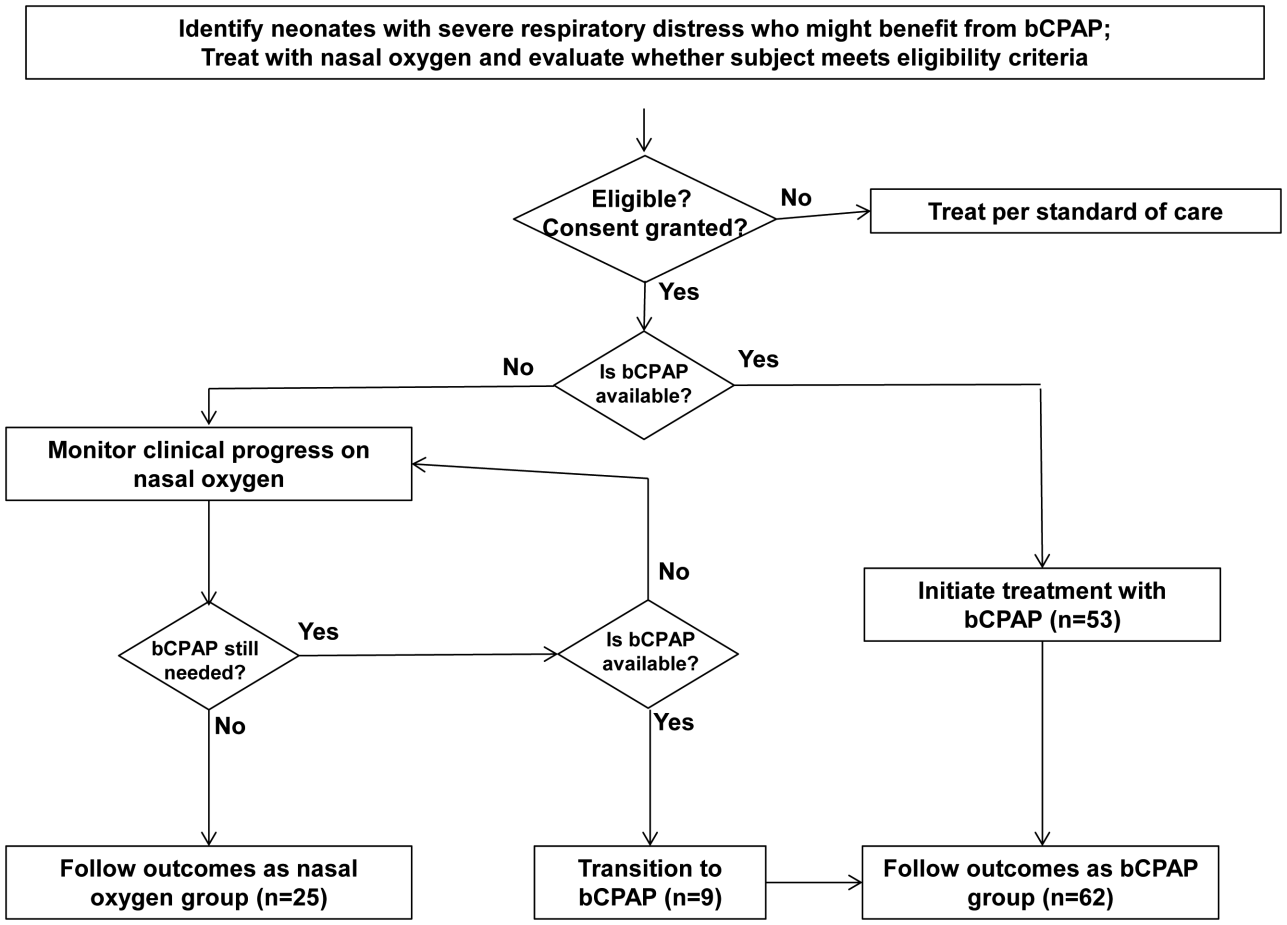
Flow chart summarizing study procedure and outcome groups.

Two low-cost bCPAP devices were installed in the ward. Figure 1 illustrates the study procedure and outcome groups. Patients were treated with bCPAP (treatment group) if a bCPAP system and trained clinical staff were available. If a bCPAP device or trained clinical staff were not available, the patient received the local standard of care, nasal oxygen (control group). This selection process was a sampling method whereby each patient was assigned to a group based on availability of a bCPAP device and appropriate staff when treatment was initiated. In some cases, a child in the control group was transitioned from nasal oxygen to bCPAP treatment after entering the study when a bCPAP device became available.

The bCPAP device has been described previously [23]. It consists of an adjustable flow generator, a pressure-regulator, and a patient interface (Fig. S1). Two pumps provide continuous flow of room air. The output of an oxygen concentrator (Airsep, New Life Intensity, 10 LPM) is connected to an input port on the device; two flow regulators adjust the flow rate and proportion of oxygen delivered. A pressure control tube submerged in a bottle of water controls end-expiratory pressure. The device delivers a mixture of pressurized air and oxygen at flow rates ranging from 0–10 L/min, pressures varying from 5–8 cm H_2_O, and oxygen ranging from 21–65%. The pumps are designed to operate for two years, and repair involves simple replacement of a US $0.50 diaphragm.

The bCPAP delivered a pressurized air mixture via Hudson bi-nasal prongs that were attached to a stockinette hat using safety pins and elastic bands (Fig. S1). Nursing care for children receiving bCPAP included twice daily suctioning to clear the airways of mucus. Sterile nasal saline drops were administered every four hours to reduce mucosal drying. Nasal oxygen was delivered from an oxygen concentrator (Airsep, New Life Intensity, 10 LPM) via standard nasal cannulae. The flow rate was set using a flow regulator and typically varied from 1–2 L/min.

Bubble CPAP and oxygen were administered until the treating clinician determined that therapy was no longer necessary. All patient care, with the exception of respiratory support, was the same for the bCPAP and standard-of-care groups. Information, excluding personal identifiers, was recorded by clinical personnel on a standardized patient monitoring form and included age, date of birth, presumed diagnosis, gender, weight, HIV status, vital signs, mode and tolerance of feeding, physical examination and method and duration of respiratory support. Vital signs were repeated one hour after recruitment (control group) or commencing bCPAP (treatment group), and twice daily afterward until discharge or death. Patients were monitored for progress and complications.

A study physician (K.K.) reviewed every child's chart for study eligibility. Each participant was assigned a final primary diagnosis of respiratory distress syndrome (RDS), congenital pneumonia, acquired pneumonia, meconium aspiration, transient tachypnoea of the newborn (TTN), or stridor based on standard clinical criteria. Co-morbidities, including sepsis and jaundice, were also noted.

### Statistical analysis

We planned to recruit a total of 50 patients in the treatment arm, matched 1∶1 with controls, giving a total sample size of 100. This would, using a one-sided Fisher's exact test, achieve at least 85% power to detect at least a 30% mortality reduction with bCPAP treatment compared with oxygen therapy with type I error = 0.05. We assumed mortality rates respectively in the treatment and control groups of 40% and 70%. To allow for potential loss due to data collection errors, we planned to enroll up to 110 patients.

Data were recorded on paper forms, then entered into Excel and ported to SPSS for analysis.

We compared demographic data, primary diagnosis, co-morbidities, and vital signs at entry for the two groups. A two-sided t-test for equality of means was performed (equal variances not assumed) to determine whether differences in continuous variables were statistically significant; for categorical variables, a two-sided Fisher's exact test was performed. Results were considered significant at the 5% level.

We calculated the survival rate with 95% confidence intervals (CIs) for the two groups. The hypothesis that the rate of survival was higher for babies receiving bCPAP than in the control group was tested using a one-sided Fisher's exact test. Logistic regression was used to calculate the odds ratio for survival with 95% CIs for babies receiving bCPAP compared to standard care. Kaplan-Meier survival curves were calculated to show the probability of survival following time from treatment initiation for the two groups.

Univariate logistic regression analysis was performed to explore whether demographic and clinical covariates were related to survival for all eligible participants. Results were considered significant at the 5% level using a two-sided Fisher's exact test for categorical variables and a t-test for continuous variables.

Subgroup analysis was conducted for three of the covariates that were related to survival (RDS, sepsis, VLBW). For each subgroup, the hypothesis that survival was higher for babies receiving bCPAP than in the control group was tested using a one-sided Fisher's exact test. Logistic regression was used to calculate the odds ratio for survival with 95% CIs for babies receiving bCPAP compared to standard care. The subgroup analyses were not defined *a priori*.

Multiple logistic regression analysis was used to calculate the survival rates, odds ratio for survival and associated 95% CIs for babies receiving bCPAP compared to standard care, adjusted for differences in baseline values of RDS, sepsis, VLBW.

## Results

Eighty-nine neonates were enrolled from January to October 2012 (Table 1). Chart review identified two neonates who did not meet eligibility criteria because they suffered severe birth asphyxia. Of the 87 eligible participants, 62 were treated with bCPAP therapy and 25 received oxygen therapy. The 62 neonates receiving bCPAP included nine who were initially assigned to the control group, but ultimately received bCPAP therapy when a device became available. Data were analysed for all 87 eligible neonates.

**Table 1:** Number of study participants and demographic data for subjects meeting eligibility criteria.

Demographic and Clinical Covariates	Treatment Group: Nasal Oxygen	Treatment Group: bCPAP	
		Transitioned from Nasal Oxygen to bCPAP	bCPAP
**Number of study participants**			
Number of subjects completing study	25	9	55
Number of subjects meeting eligibility criteria	25	9	53
**Gender**			
% Male	60.0%	44.4%	60.4%
% Female	40.0%	55.6%	39.6%
**Gestational Age**			
Average (weeks)	33.0 weeks	32.0 weeks	33.2 weeks
Unknown (%)	12.0%	0.0%	9.4%
**Average Birth Weight (kg)**	**1.68 kg**	**1.37 kg**	**1.79 kg**
Very Low Birth Weight (> = 1.0 kg–<1.5 kg) (%)	52.0%	41.5%	77.8%
Low Birth Weight (> = 1.5 kg–<2.5 kg) (%)	28.0%	41.5%	22.2%
Birth Weight > = 2.5 kg (%)	20.0%	17.0%	0.0%
**Location of birth**			
Queen Elizabeth Central Hospital (QECH) (%)	60.0%	55.6%	56.6%
Outside QECH (%)	32.0%	44.4%	32.1%
Unknown (%)	8.0%	0.0%	11.3%
**Singletons vs. Multiples**			
Singletons (%)	52.0%	44.5%	73.6%
Multiples (%)	44.0%	44.4%	26.4%
Unknown (%)	4.0%	11.1%	0.0%
**Received bag & mask ventilation prior to therapy?**			
Yes (%)*	4.0%	22.2%	24.5%
No (%)	88.0%	66.7%	54.7%
Unknown (%)	8.0%	11.1%	20.8%
**HIV Status**			
Exposed	20.0%	33.3%	22.7%
Unexposed	64.0%	44.4%	67.9%
Unknown	16.0%	22.2%	9.4%
**Entry vital statistics**			
Average entry heart rate (beats per minute (bpm))	144 bpm	148 bpm	141 bpm
Average entry respiratory rate (bpm)	54 bpm	55 bpm	54 bpm
Average entry oxygen saturation (%)	92%	91%	88%
**Primary Diagnosis**			
RDS	68.0%	100.0%	73.6%
Congenital Pneumonia	4.0%	0.0%	18.9%
Acquired Pneumonia	4.0%	0.0%	0.0%
Meconium Aspiration	8.0%	0.0%	5.7%
Transient Tachypnea of the Newborn	12.0%	0.0%	0.0%
Stridor	4.0%	0.0%	0.0%
Asphyxia	0.0%	0.0%	1.8%
**Co-Morbidities**			
Sepsis	28.0%	44.4%	41.5%

* Difference in group receiving nasal oxygen and bCPAP significantly different (p = 0.015).

Table 1 summarizes the demographic and clinical covariates for the eligible neonates. Differences in the mean values of covariates for the group receiving bCPAP were not statistically significant compared to those for the control group with one exception: the fraction of babies who required bag and mask ventilation before recruitment was higher in the bCPAP group than in the control group (p = 0.015). Most participants had very low birth weight (VLBW) and were born at QECH. Over 25% of neonates in each group were from multiple births, and approximately 20% of neonates in each group were exposed to HIV.

**Figure 2:**
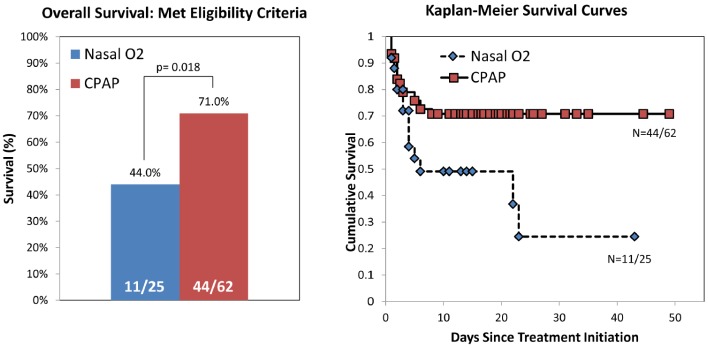
Overall survival of eligible study participants receiving nasal oxygen vs. bCPAP. (Left) Fraction of eligible study participants who survived to discharge and received nasal oxygen or bCPAP. When treated with bCPAP, the survival rate of infants with severe respiratory distress is significantly higher than for those treated with nasal oxygen (p = 0.018). Without adjustment, bCPAP is associated with a 3.1-fold increase in odds of survival (confidence interval 1.2–8.1, significance = 0.02). (Right) Kaplan-Meier survival curves showing cumulative survival vs. days since treatment initiation for infants with severe respiratory distress treated with bCPAP (n = 62) and those treated with nasal oxygen (n = 25).

Figure 2 compares survival to discharge in the two groups. The survival rate in the control group was 44.0% (95% CI: 26–63%); survival in the bCPAP group was 71.0% (95% CI: 59–81%) (p = 0.018). Without adjustment, bCPAP is associated with a 3.1 fold increase in odds of survival (95% CI: 1.2–8.1, p = 0.02). Figure 2 also shows Kaplan-Meier survival curves of cumulative survival vs. time following initiation of treatment. These are different by the log rank test at p = 0.013.

Univariate logistic regression analysis indicated that five of the covariates in Table 1 were related to survival: a primary diagnosis of RDS (p = 0.002); co-morbidity of sepsis (p = 0.039); birth weight (p = 0.003); a birth weight in the VLBW range (p = 0.016); and gestational age (p = 0.023). Further subgroup analyses were performed by birth weight, presence of RDS and of sepsis. As gestational age was not available for approximately 10% of subjects, no subgroup analysis was conducted for this variable.

**Figure 3:**
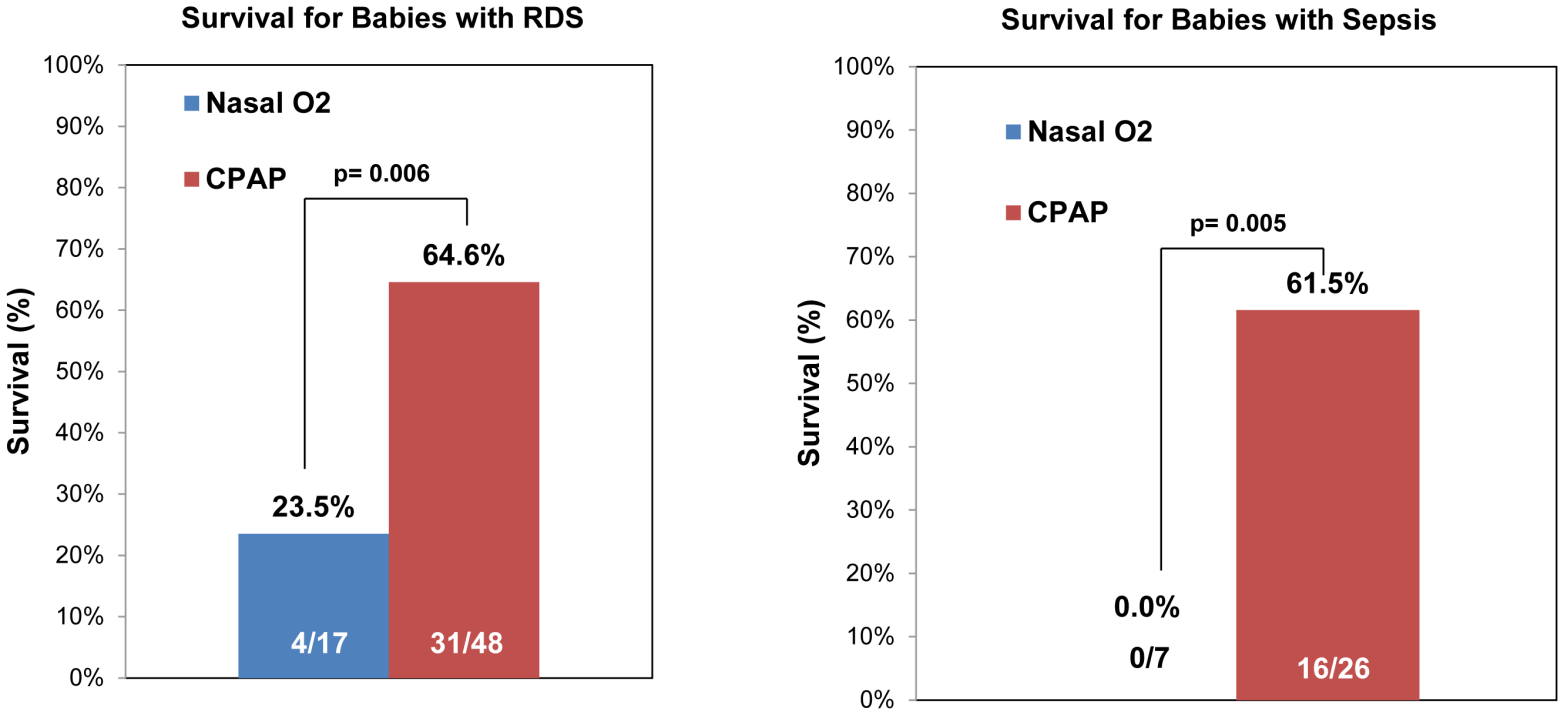
Survival of participants with RDS and sepsis receiving nasal oxygen vs. bCPAP. (Left) Fraction of eligible study subjects with a primary diagnosis of respiratory distress syndrome (RDS) who survived to discharge and received nasal oxygen or bCPAP. (Right) Fraction of eligible study subjects with a co-morbidity of sepsis who survived to discharge and received nasal oxygen or bCPAP.

Figure 3 compares survival for eligible neonates with a primary diagnosis of RDS and those with sepsis. For neonates with RDS, survival was 23.5% (95% CI: 9–49%) in the control group, compared to 64.6% (95% CI: 50–77%) in the bCPAP group (p = 0.006). None of the seven neonates with sepsis in the control group survived, while survival was 61.5% (95% CI: 42–78%) in the bCPAP group (p = 0.005).

**Figure 4:**
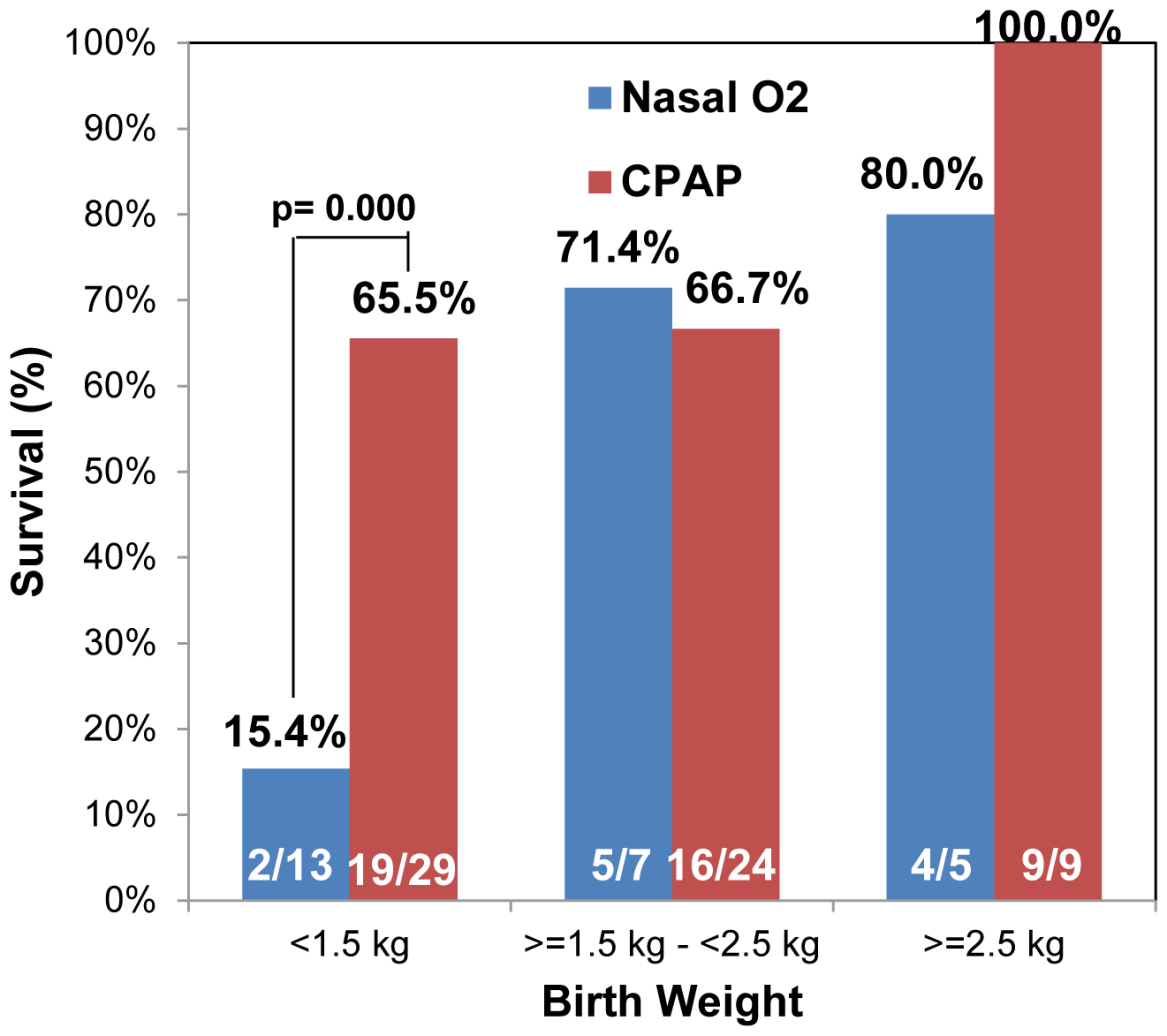
Survival of participants by birthweight receiving nasal oxygen vs. bCPAP. Fraction of eligible study subjects who survived to discharge and received nasal oxygen or bCPAP, stratified by birth weight. Results are reported for subjects with very low birth weight (> = 1.0 kg to <1.5 kg), low birth weight (> = 1.5 kg to <2.5 kg) and birth weights greater than or equal to2.5 kg.

Figure 4 shows survival rates for eligible neonates in the two groups, stratified by birth weight. Improvements in survival are greatest for VLBW infants (≥1.0–<1.5 kg); in whom survival was 15.4% (95% CI: 4–45%) in the control group compared to 65.5% (95% CI: 47–80%) in the bCPAP group (p≤0.001). For babies with low birth weight (≥1.5 kg–<2.5 kg) and babies with birth weight ≥2.5 kg, differences in survival were not statistically different for the two groups.

Multiple logistic regression analysis was performed to adjust for baseline differences in RDS, sepsis, and VLBW; the adjusted survival rate for babies receiving bCPAP is 85% (95% CI: 69–94%), while that for standard care is 47% (95% CI: 23–73%) and bCPAP is associated with an adjusted 6.5 fold increase in odds of survival (95% CI: 1.9–22.7, p = 0.003).

Table 2 compares the secondary outcomes of treatment time and duration of hospital stay for all eligible subjects and for those who survived to discharge. On average, neonates receiving bCPAP spent five more days in the hospital and nearly three more days on treatment than those in the control group. On average, neonates who were transitioned from nasal oxygen to bCPAP spent twice as long in the hospital and twice as long on treatment as those who received bCPAP initially; the average delay in receiving bCPAP was 3.1 days. Similar trends in increased treatment and hospitalization time were seen for neonates who survived to discharge. The increase in treatment and hospitalization time in bCPAP could be explained by the differences in primary diagnosis between the two groups; children receiving bCPAP were more likely to suffer from RDS than those receiving nasal oxygen.

**Table 2:** Average duration of treatment and hospital stay (+/− standard deviation) for eligible subjects and survivors versus treatment group.

All eligible neonates	Nasal Oxygen	bCPAP	Transitioned from nasal oxygen to bCPAP
Average hospital stay (days)	9.10 (9.70)	14.13 (10.87)	22.00 (14.14)
Average time from study entry to treatment with bCPAP (days)	NA	0.19 (0.42)	3.11 (3.18)
Average total time on treatment (days)	4.00 (4.37)	6.91 (5.37)	13.94 (11.45)
Average time receiving nasal oxygen (days)	4.00 (4.37)	1.84 (3.04)	9.83 (9.68)
Average time receiving bCPAP (days)	NA	5.07 (3.66)	4.11 (2.87)

Survivors	Nasal Oxygen	bCPAP	Transitioned from nasal oxygen to bCPAP
Average hospital stay (days)	13.27 (11.00)	18.89 (9.60)	27.57 (10.83)
Average time from study entry to treatment with bCPAP (days)	NA	0.27 (0.49)	3.86 (3.24)
Average total time on treatment (days)	3.59 (3.05)	8.69 (5.41)	17.36 (10.77)
Average time receiving nasal oxygen (days)	3.59 (3.05)	2.58 (3.36)	12.43 (9.50)
Average time receiving bCPAP (days)	NA	6.11 (3.72)	4.93 (2.73)

Only mild complications were associated with bCPAP (Table S1), including nasal irritation, facial irritation, and epistaxis. Similar complication rates were observed in the control group. No clinical diagnoses of pneumothorax were made, but as routine x-rays and cranial ultrasound were unavailable, silent complications cannot be ruled out.

## Discussion

This study demonstrates the potential impact of introducing a low-cost, appropriate bCPAP system in a low-resource setting where standard therapy is nasal oxygen. Using bCPAP to treat newborns with severe respiratory distress resulted in a 27% absolute improvement in survival to discharge. The benefit was more pronounced in neonates with VLBW, RDS, or sepsis. Only 24% of neonates with RDS treated with nasal oxygen survived to discharge, compared to 65% receiving bCPAP, reflecting similar rates of survival as observed in the US during the transition from nasal oxygen to bCPAP for treatment of RDS [5].

The value of CPAP therapy for neonatal RDS is well-accepted in high-resource settings but very few studies have examined the value of CPAP therapy in resource-limited settings lacking advanced respiratory support [16], [20], [29]. These studies have either included older children [16], examined immediate improvements in clinical endpoints rather than survival to hospital discharge [16], or been small, observational studies [20], [29].

Our study was carried out in a typical low-resource setting using a bCPAP that was conceived, designed and evaluated with input from local physicians and nurses, ensuring that the device addresses competencies and capacity of this under-resourced environment [23], The technical performance of the device was monitored throughout the study. Therapeutic flow and pressure met performance standards reliably without preventive maintenance, and no device failures occurred. In contrast, 40% of study oxygen concentrators failed during this same period when circuit boards were damaged by line voltage spikes. To meet cost and infrastructure constraints, the bCPAP device did not heat or humidify the mix of pressurized air and oxygen delivered via the nasal prongs. Instead, humidification was provided through routine use of nasal saline drops. This did not appear to result in significant complications (Table S1).

Our study has a number of limitations. As bCPAP is known to be an effective therapy [5], [6], [8], we did not perform a randomised trial. Instead, the decision to treat with bCPAP was based on availability of a bCPAP device. This design allowed potential bias. We planned to recruit equal numbers of patients in each arm to detect a difference between the two groups. However, since allocation of CPAP to eligible subjects was based on device availability actual enrollment was dependent on available equipment and staffing resources. Our original estimate was that CPAP devices would be available approximately half the time needed. To allow for potential loss due to data collection errors, we planned to enroll up to 110 patients. In practice, CPAP devices were available more frequently than not and thus more study participants received CPAP than nasal oxygen. The study was stopped when over 60 participants received CPAP; at this point, the projected number of CPAP patients had been enrolled plus the entire additional allowed margin to account for data loss.

In addition, this design could result in treating infants with more severe illness with bCPAP and those with less severe illness with nasal oxygen. Indeed a significantly higher proportion of infants treated with bCPAP required resuscitation prior to therapy (24.5%) than those who received nasal oxygen (4.0%). We monitored the status of children receiving nasal oxygen. Nine of them were transitioned to bCPAP when they failed to improve and a device became available. The outcomes of these nine children were analysed with the bCPAP group. All of the babies in the CPAP group received CPAP within 36 hours of being identified as needing CPAP, with a majority receiving it immediately. Five of the nine babies in the group that transitioned to CPAP also received CPAP treatment within 36 hours of being identified as needing CPAP. Because, in general, preterm infants who survive the first few postnatal days have an increased chance of long-term survival, transitioning babies into the CPAP group after a few days in the oxygen group might have created bias in favour of CPAP.

To account for this potential bias, we performed a per-protocol analysis, comparing outcomes for infants who received bCPAP to those who received nasal oxygen; babies who initially received oxygen but were later transitioned to CPAP are not included in the per-protocol analysis. The survival rate in the control group was 44.0%; survival in the bCPAP group was 69.8% (p = 0.014). For neonates with RDS, survival was 23.5% in the control group, compared to 61.5% in the bCPAP group (p = 0.004). Again, improvements in survival were found to be greatest for VLBW infants (≥1.0–<1.5 kg); in whom survival was 15.4% in the control group compared to 57.1% in the bCPAP group (p = 0.006).

Analysing these nine babies as part of the standard care group, analogous to intent-to-treat, we find that 69.8% (37/53) of children receiving bCPAP survived to discharge, compared to 52.9% (18/34) for standard care; the improvement in survival approaches significance (p = 0.087). Finally, we did not determine the optimal time to initiate or terminate treatment with bCPAP. Additional studies are needed to determine if earlier commencement of bCPAP could further improve outcomes in this setting.

Neonatal deaths account for 41% of global child mortality; the neonatal mortality rate has changed little in the last decade [30]. If our results are generalizable, we estimate that on the African continent, where nearly one million babies die each year within a week of birth [31], providing low-cost bCPAP in central and district hospitals could prevent 178,000 neonatal deaths. While the cost of the bCPAP device has been reduced, the cost and availability of consumables, staff support and support equipment remain a barrier to scale-up. Nonetheless, implementing such a system has the potential to improve neonatal care and health outcomes in low-resource settings.

## Supporting Information

Figure S1 **Photograph showing bubble CPAP device used in the study.** bCPAP was delivered using Hudson prongs secured to a stretchy hat with safety pins and elastic bands. The bCPAP delivered a blended mix of air and oxygen from an oxygen concentrator at flow rates varying from 0–10 L/min and pressures ranging from 5–8 cm H_2_O.
(TIF)

Table S1 **Fraction of eligible participants in each treatment group who experienced minor complications during treatment.**
(PDF)
