# Supplementary figures and images for: Efficacy of a Low-Cost Bubble CPAP System in Treatment of Respiratory Distress in a Neonatal Ward in Malawi
Source: PLoS One. 2014 Jan 29;9(1):e86327. doi: 10.1371/journal.pone.0086327 (PMC3906032; doi:10.1371/journal.pone.0086327)

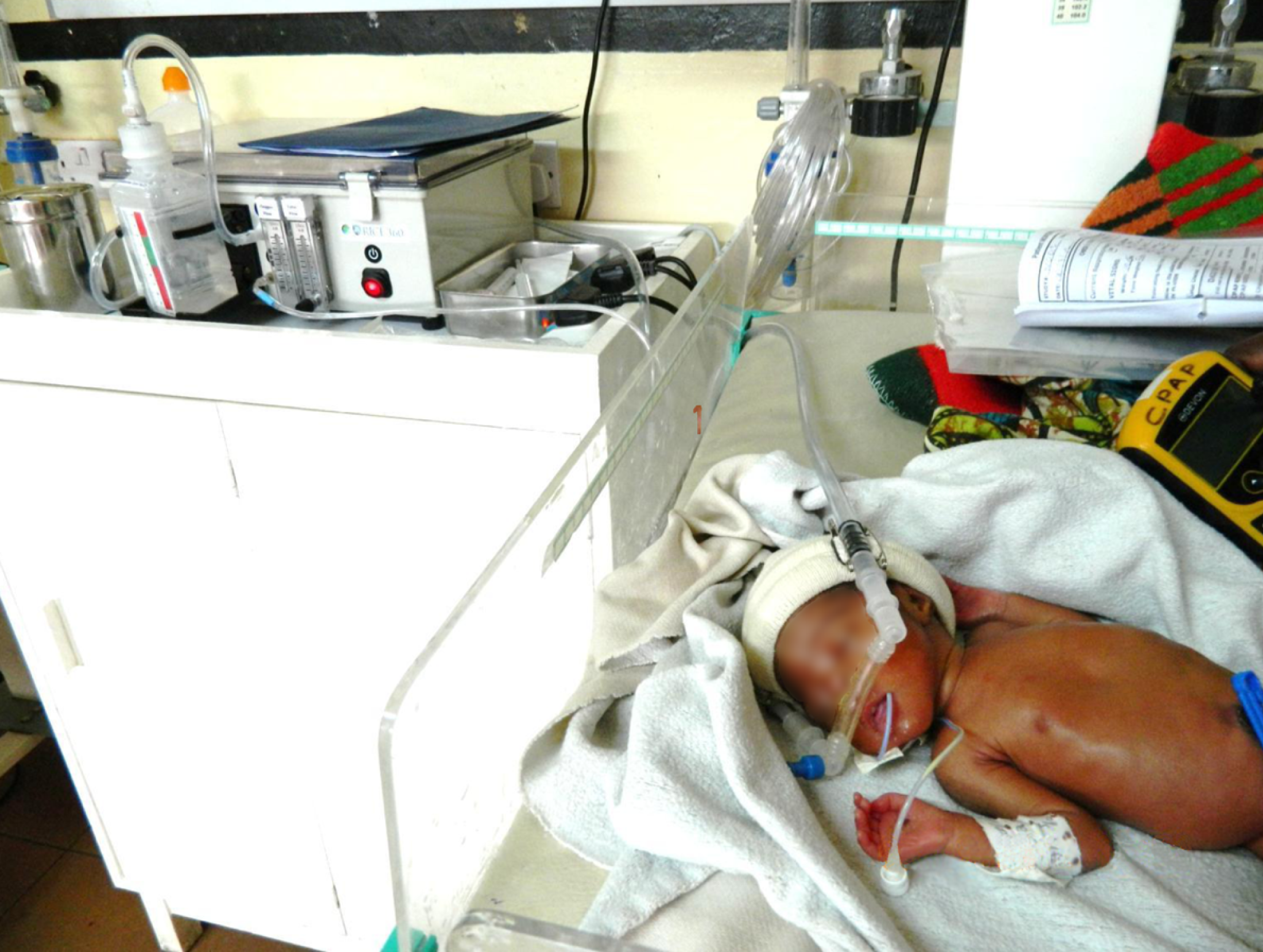

Supplement: Figure S1 — Photograph showing bubble CPAP device used in the study. bCPAP was delivered using Hudson prongs secured to a stretchy hat with safety pins and elastic bands. The bCPAP delivered a blended mix of air and oxygen from an oxygen concentrator at flow rates varying from 0–10 L/min and pressures ranging from 5–8 cm H2O. (TIF) [file pone.0086327.s001.tif]
