# Supplementary material for: Efficacy of a Low-Cost Bubble CPAP System in Treatment of Respiratory Distress in a Neonatal Ward in Malawi
Source: PLoS One. 2014 Jan 29;9(1):e86327. doi: 10.1371/journal.pone.0086327 (PMC3906032; doi:10.1371/journal.pone.0086327)
Supplement: Table S1 — Fraction of eligible participants in each treatment group who experienced minor complications during treatment. (PDF) [file pone.0086327.s002.pdf]

Table S1: Fraction of eligible participants in each treatment group who experienced minor complications during treatment.

**Complication**	Nasal Oxygen	bCPAP	Transitioned from nasal oxygen to bCPAP
Mild facial irritation, < 1 day duration	3 (12%)	1 (1.9%)	0 (0.0%)
Mild nasal irritation, <3 day duration	1 (4.0%)	7 (13.2%)	2 (22.2%)
Mild epistaxis	1 (4.0%)	6 (11.3%)	2 (22.2%)
